# Application of genetic testing criteria for hereditary breast cancer in South Africa

**DOI:** 10.1007/s10549-024-07585-3

**Published:** 2025-01-07

**Authors:** T. S. Osler, M. Schoeman, W. J. S. Pretorius, C. G. Mathew, J. Edge, M. F. Urban

**Affiliations:** 1 Sydney Brenner Institute for Molecular Bioscience and Division of Human Genetics, Faculty of Health Sciences, University of the Witwatersrand, Johannesburg, South Africa; 2 Division of Human Genetics, Faculty of Health Sciences, National Health Laboratory Service and University of the Witwatersrand, Johannesburg, South Africa; 3 Division of Molecular Biology and Human Genetics, Faculty of Medicine and Health Sciences, University of Stellenbosch and Tygerberg Hospital, Parow, South Africa; 4 Department of Surgery, Charlotte Maxeke Johannesburg Academic Hospital and University of the Witwatersrand, Johannesburg, South Africa; 5 Department of Obstetrics and Gynaecology, Faculty of Medicine and Health Sciences, University of Stellenbosch and Tygerberg Hospital, Parow, South Africa

**Keywords:** Hereditary breast cancer, *BRCA1/2*, Testing guidelines, Family history, LMIC

## Abstract

**Purpose:**

Breast cancer (BC) is the commonest cancer in South African women. A proportion are associated with a pathogenic or likely pathogenic (P/LP) variant in a BC susceptibility gene. Clinical guidelines for genetic testing are used to optimise variant detection while containing costs. We assessed the detection rate in women of diverse ancestries who met the South African National Department of Health (NDOH) testing guidelines, and analysed relationships between testing criteria, participant characteristics and presence of a *BRCA1/2* P/LP variant.

**Methods:**

Records from 376 women with BC who met NDOH criteria and had genetic testing were included. Demographic, clinical and test result data were collated to describe detection rates according to criteria met, and a multivariate analysis conducted to find variables most frequently associated with a P/LP variant.

**Results:**

P/LP variant prevalence in women meeting NDOH testing criteria was 19.9% (75/376). Women meeting ≥ 2 guideline criteria were over twice as likely to have a P/LP variant (OR 2.27, 95%CI 1.27–4.07, *p* = 0.006), highlighting the guidelines’ capacity to stratify risk. Family history (OR 1.97; 95%CI 1.05–3.70, *p* = 0.03) and Black African ancestry (OR 2.58; 95%CI 1.28–5.18, *p* < 0.01) were independently associated with having a *BRCA1/2* P/LP variant when controlling for other variables. Notably, although Black African participants were less likely to report a family history, those that did had higher odds of a P/LP variant in *BRCA1/2*.

**Conclusion:**

These results demonstrate the usefulness of the NDOH guidelines in women of diverse ancestries and provide insight into the factors associated with P/LP variants in understudied African populations.

**Supplementary Information:**

The online version contains supplementary material available at 10.1007/s10549-024-07585-3.

## Introduction

There were 11,020 women diagnosed with breast cancer (BC) in South Africa in 2022, making it the commonest cancer in females [1]. A proportion are expected to be attributable to pathogenic or likely pathogenic (P/LP) variants in a germline BC susceptibility gene, such as *BRCA1/2.* Population-based studies from the USA put this proportion at 5–10% [2, 3]. Similar data from Africa is scarce, but West African studies give estimates of 8–14% [4–6]. P/LP variants in these genes are associated with lifetime risks of BC of 40%–85% for high risk genes and 20%–40% for moderate risk genes, causing significant morbidity and mortality [7]. In addition, the risks of other cancers, such as ovarian and pancreatic cancers, are often also increased in gene carriers. Identifying individuals with these P/LP variants allows intensified surveillance, risk-reducing surgical interventions and modifications to cancer treatment to be made [8].

Given the modest proportion of BC associated with Mendelian susceptibility genes, and the historically high cost of genetic testing, clinical guidelines were developed to direct testing [9, 10]. Guidelines initially aimed to identify individuals with a 10%–20% risk of carrying a germline P/LP variant in a high risk BC susceptibility gene, such as *BRCA1/2, TP53* and *PALB2* [7]. Following the introduction of extended gene panel testing it has been noted that these guidelines are less successful at identifying individuals with P/LP variants in moderate penetrance genes, such as *ATM* and *CHEK2* [11]. As a result of this, the increasing availability of targeted therapies and the decreasing cost of genetic testing over time, many high-income countries (HIC) have adjusted their policy guidelines to reduce the stringency of genetic testing criteria, with some calling for universal testing of women with BC [10, 12]. In low- and middle-income countries (LMIC) the successful implementation of genetic testing for hereditary cancer faces technological, health system, and patient-level barriers [13]. Clinical practice guidelines are necessary for ensuring accessible genetic testing and are recognised as a key need in some LMICs [14]. Although guidelines have been published in a few LMICs [15, 16] there is limited data available on their usefulness [13].

In 2018, the South African National Department of Health (NDOH) published guidelines to establish standards for BC prevention and control. This included eligibility criteria for genetic testing, which remain in effect [17]. These criteria were adapted from the widely used National Comprehensive Cancer Network (NCCN) guidelines from the USA namely, “Genetic/Familial High-Risk Assessment: Breast and Ovarian, version 2.2016”, which have since been updated [18]. Criteria used in the NDOH and NCCN guidelines are summarised in Table 1 and show that the NDOH criteria were made more stringent by lowering the eligible age at diagnosis. Minimal local data was available to guide this process.

**Table 1:** A comparison of NDOH and NCCN v2.2016 criteria used to select women with breast cancer for genetic testing

Genetic testing criteria for women with breast cancer	NDOH^a^ criteria [36]	NCCN^b^ v2.2016 criteria [18]
Known P/LP variant in a cancer predisposition gene in the family	Yes	Yes
Age of breast cancer diagnosis	≤ 40 years	Early-age onset BC (suggested ≤ 50y)
Triple negative breast cancer^c^ < 60 years	Yes	Yes
Two primary breast cancers at any age	Yes	Yes
≥ 1 close relatives with breast cancer < 50 years	Yes	Yes
≥ 1 close relative/s with invasive ovarian cancer	Yes	Yes
≥ 1 close relative/s with male BC	Yes	Yes
≥ 2 close relatives with breast/pancreatic cancer with at least one < 60 years old	Yes	Yes
From a population at increased risk	Absent	Yes

^a^NDOH – National Department of Health (South Africa)

^b^NCCN – National Comprehensive Cancer Network (USA)

^c^Triple negative breast cancer—cancers that are estrogen and progesterone hormone receptor negative and human epidermal growth factor receptor 2 (HER2) amplification negative

The suitability of these guidelines in our setting is uncertain for several reasons. The NCCN guidelines were developed using data from high-resource settings with predominantly European ancestry populations [19]. In contrast, South Africa is an upper middle income country with a majority Black African population [20]. Incidence rates of BC vary considerably in different countries and populations. In South Africa, the age standardised incidence rate per 100 000 of BC in 2022 was 94.6 in White females (similar to European populations), 47.5 in Mixed Ancestry females and 21.2 in Black African females [1, 21]. These differences raise uncertainty about whether similar genetic factors contribute to BC risk across the populations. In addition, the predictiveness of individual criteria is uncertain. For instance, one criterion is the presence of a breast tumour before the age of 60 that is estrogen and progesterone receptor negative with no overexpression of human epidermal growth factor receptor 2 (HER2), referred to as triple negative breast cancer (TNBC). Notably, populations with African ancestry may have a higher prevalence of TNBC [22, 23]. Although TNBC has been shown to be associated with *BRCA1* P/LP variants in Nigerian patients [5], African American women with TNBC are less likely to have a P/LP variant in *BRCA1* compared to women from other ancestry groups [24]. Consequently, the positive predictive value of TNBC for identifying P/LP variants in African populations remains unclear. Furthermore, Black African women in South Africa have on average a younger age of diagnosis than White women, [25] which suggests that the age of BC diagnosis criterion may be less predictive of a P/LP variant in Black African women. Whether the NDOH guidelines are an effective tool for detecting P/LP variants in BC genes in South African women is unknown.

In South Africa, there is tension between optimising the detection rate of BC susceptibility genes and the cost of testing. The objectives of this study were to determine the P/LP variant prevalence in BC susceptibility genes in women meeting the NDOH guidelines in a South African setting and to compare the detection rates of individual criteria. In addition, we conducted multivariate analysis to assess the association of various demographic, clinical and tumour histological features with *BRCA1/2* detection rates.

## Methods

### Participants

Participants included females with invasive or in situ BC who attended genetic counselling and testing for hereditary BC between 1 January 2018 and 1 October 2022 at Tygerberg Academic Hospital (TAH) in the Western Cape, South Africa. Participants were included if they met the NDOH testing criteria as shown in Table 1.

Of the 424 women who had testing during this period, 26 individuals were excluded from this study because they did not meet NDOH criteria. Additionally, 13 participants were excluded because they tested negative for common founder variants in *BRCA1/2* but were not tested for the presence of other potential P/LP variants in *BRCA1/2*. There were 9 participants with leiomyosarcoma, ovarian or pancreatic cancer who were excluded, as although these cancers are associated with P/LP variants in BC susceptibility genes, they are not included in the NDOH criteria. The remaining 376 participants were included in the study to determine P/LP variant prevalence. Three participants with a known familial P/LP variant were excluded from the multivariate analyses, as this indication is associated with a substantially higher likelihood of having a P/LP variant compared to other NDOH criteria.

### Data collection

Demographic, clinical, and test result data were collected for participants from TAH genetic counselling and histology records. Information on proband ancestry, required by the genetic testing laboratory to assist in variant interpretation, was inferred by the genetic counsellor in keeping with the population group categories of the South African National Census of 2022; White, Black African or Coloured. The latter descriptor is referred to as Mixed Ancestry in this study as the population has various ancestral roots, notably Khoe-San, European, Black African, and South and South-East Asian [26]. For all variables described in the study, data was available for > 97% of patients.

### Genetic testing

During the study period, genetic testing for hereditary BC was done at Invitae Corporation [27] in the USA in 90.4% (340/376) of participants, with 12.8% (48/376) of participants tested locally at the National Health Laboratory Service (NHLS) [28]. Although participants had testing of between 2 and 102 genes, we included only the results for 13 genes (*ATM, BARD1, BRCA1, BRCA2, CDH1, CHEK2, NF1, PALB2, PTEN, RAD51C, RAD51D, STK11, TP53)* known to be associated with BC and having clinical guidelines for risk-reduction. Because not all probands had testing of the same genes, with the exception of *BRCA1/2*, denominators used to calculate the prevalence of P/LP variants differed. In addition, it meant we could not include the detection of P/LP variants in genes other than *BRCA1/2* in the multivariate analysis of NDOH criteria.

The Invitae gene panel protocol used next-generation sequencing (NGS) to detect copy number and single nucleotide variants in coding exons and 10–20 flanking base pairs for most genes [27, 29]. The Sherloc modifications of the American College of Genetics and Genomics and the Association for Molecular Pathology (ACMG-AMP) guidelines were used by Invitae for variant interpretation [30]. NHLS genetic testing was done using various methods: targeted analysis of 8 well-documented founder pathogenic variants in *BRCA1/2*; single nucleotide and copy number variant (CNV) detection within the exons and splice-site junctions of *BRCA1/2*; family variant testing; and a next generation sequencing (NGS) panel of 15 genes [31]. NHLS testing was conducted in a diagnostic laboratory accredited by the South African National Accreditation System (SANAS) [32], with variants interpreted using the ACMG-AMP variant interpretation guidelines [33].

### Data analysis

Study data were stored in a RedCap Database [34] and statistical analysis was done using RStudio [35]. A *P* value < 0.05 was considered statistically significant. Chi-square or Fischer’s exact test were used to find group differences when variables were categorical and the Kruskal–Wallis test when data was continuous. When significant differences were found, odds ratios, 95% confidence intervals and associated p values were calculated.

Logistic regression was used to examine the relationship between detecting a P/LP variant in *BRCA1/2* and demographic and clinical variables, as well as NDOH criteria. Initially, each variable was included in a univariate model to determine its predictive value (Table 2). Variables with *p* < 0.2 were included in the multivariate analysis. The absence of multicollinearity between the predictor variables was confirmed using variance inflation factors. As ancestry was comprised of three categories, the Mixed Ancestry group was used as the reference/index category with White and Black African groups compared to it. Results are reported as odds ratios (OR) with 95% confidence intervals (CI) and associated p-values.

**Table 2:** Pathogenic/Likely Pathogenic variant detection rates by NDOH testing criteria

No. NDOH criteria met (*n* = 376)	Testing criteria	No. proband	Gene with P/LP variant detected^a^						Total detection rate in all genes^f^
			*BRCA1/2*	*ATM*	*CHEK2*	*PALB2*	*TP53*	*RAD51C*	
	Known variant in the family	3	2/3 (66.7%)						66.7%
One criterion	Family history	58	8/58 (13.8%)	4/56 (7.1%)	1/56 (1.8%)				22.9%
	TNBC^b^ at < 60 years	73	8/73 (11.0%)					1/57 (1.8%)	12.7%
	Age of diagnosis ≤ 40	119	14/119 (11.8%)	2/109 (1.8%)	2/109 (1.8%)	1/110 (0.9%)			16.3%
	> 1 primary BC	15	1/15 (6.7%)			1/12 (8.3%)			15.0%
Two criteria^c^		90	19/89 (21.3%)			2/85 (2.4%)	2/85 (2.4%)		26.1%
Three criteria^c^		18	6/18 (33.3%)					1/15 (6.7%)	40.0%

^a^Denominators reflect the number of participants who had testing for the gene in question,

^b^TNBC refers to triple negative breast cancer which is estrogen and progesterone receptor negative and HER2 amplification negative,

^c^Indications include any combination of the following criteria: family history, triple negative breast cancer, young age of diagnosis and > 1 primary BC. Known variant in the family was not included in this analysis

^d^Comparison of *BRCA1/2* P/LP variant prevalence in participants meeting only one of the four criteria (family history, triple negative breast cancer, age at diagnosis, > 1 primary BC)

^e^Comparison of prevalence of P/LP variants in other BC genes in participants meeting one of the four criteria (family history, triple negative breast cancer, age at diagnosis, > 1 primary BC)

^f^Calculated by summing percentages in the preceding columns

## Results

The prevalence of P/LP variants found in the 376 study participants was 19.9% (75/376), with 17.0% in high-penetrance (*BRCA1/2, PALB2, TP53*) and 2.9% in moderate penetrance (*ATM, CHEK2, RAD51C, RAD51D*) genes. 15.4% (58/376) of the participants had a P/LP variant in *BRCA1/2*, with more in *BRCA2* (35/376, 9.3%) than in *BRCA1* (23/375, 6.1%) (see Supplementary Table 1 for a list of P/LP variants detected).

To assess the NDOH criteria, we first compared the prevalence of P/LP variants for each criterion (Table 2). Of participants who satisfied only one of the NDOH criteria (either family history, triple negative breast cancer (TNBC), young age of cancer diagnosis or > 1 primary BC), 11.7% (31/265) had a P/LP variant in *BRCA1/2* and 5.1% (12/235) in one of the other BC susceptibility genes. Individually, each of the four criteria was associated with a similar prevalence of P/LP variants in *BRCA1/2* (*p* = 0.88) and other BC susceptibility genes (*p* = 0.35). However, participants who met two or three of the criteria were twice as likely to have a P/LP variant detected in *BRCA1/2* (OR 2.27, 95% CI 1.27–4.07, *p* = 0.006) than those meeting only one. This increased likelihood did not apply to other BC susceptibility genes, where meeting one or more criteria resulted in similar odds of having a P/LP variant (OR 1.02, 95% CI 0.35–2.98, *p* = 0.82).

Table 3 presents the univariate analysis of test indications, demographic data and clinical variables of 373 participants (three participants with known family variant were excluded) by their *BRCA1/2* P/LP variant status. Overall, age at diagnosis was the most frequently met NDOH test criterion (55.0%), followed by TNBC (35.4%) and family history (34.3%), with > 1 primary BC (9.4%) being the least frequent. We assessed the influence of each of these four criteria, along with participant demographic and clinical variables, on the odds of detecting a P/LP variant in *BRCA1/2* using multivariate logistic regression. Based on the initial univariate analysis, we included ancestry, family history, > 1 primary BC and cancer stage at diagnosis in the logistic regression model (Table 4).

**Table 3:** Demographic and clinical data for study participants by *BRCA1/2* status

		Participants (*n* = 373)	*BRCA1/2* negative (*n* = 317)	*BRCA1/2* positive (*n* = 56)	*P* value
Demographic and clinical variables					
Ancestry/population group	Mixed Ancestry	226 (60.6%)	199/226 (88.1%)	27/226 (11.9%)	0.05*
	Black African	93 (24.9%)	74/93 (79.6%)	19/93 (20.4%)	
	White	54 (14.5%)	44/54 (81.5%)	10/54 (18.5%)	
Age at diagnosis	Median	39	39	38	0.45
	Range	18–76	18–76	24–73	
Breast cancer type^a^	Ductal carcinoma in situ	6/364 (1.6%)	5/6 (83.3%)	1/6 (16.7%)	0.36
	Invasive ductal carcinoma	323/364 (88.7%)	274/323 (84.8%)	49/323 (15.2%)	
	Invasive lobular carcinoma	14/364 (3.8%)	12/14 (85.7%)	2/14 (14.3%)	
	Other	21/364 (5.8%)	20/21 (95.2%)	1/21 (4.8%)	
Breast tumour receptors	Triple negative breast cancer	133/370 (35.9%)	113/133 (85.0%)	20/133 (15.0%)	0.94
	Other	237/370 (64.1%)	202/237 (85.2%)	35/237 (14.8%)	
Cancer stage^b^	in situ	6/368 (1.6%)	5/6 (83.3%)	1/6 (16.7%)	0.10*
	Stage 1	21/368 (5.7%)	18/21 (85.7%)	3/21 (14.3%)	
	Stage 2	127/368 (34.5%)	102/127 (80.3%)	25/127 (19.7%)	
	Stage 3	158/368 (42.9%)	140/158 (88.6%)	18/158 (11.4%)	
	Stage 4	53/368 (14.4%)	44/53 (83.0%)	9/53 (17.0%)	
	Recurrence	3/368 (0.8%)	3/3 (100%)	0	
NDOH indications					
Family history	Meets NDOH family history criterion	128/373 (34.3%)	103/128 (80.5%)	25/128 (19.5%)	0.09*
	Does not meet NDOH family history criterion	245/373 (65.7%)	214/245 (87.3%)	31/245 (12.7%)	
Triple negative breast cancer < 60 years	Triple negative breast cancer	132/373 (35.4%)	112/132 (84.8%)	20/132 (15.2%)	1.0
	Other	241/373 (64.6%)	205/241 (85.1%)	36/241 (14.9%)	
Age at diagnosis	≤ 40 years	205/373 (55.0%)	172/205 (83.90%)	33/205 (16.1%)	0.56
	> 40 years	168/373 (45.0%)	145/168 (86.3%)	23/168 (13.7%)	
> 1 primary BC	Yes	35/373 (9.4%)	26/35 (74.3%)	9/35 (25.7%)	0.08*
	No	338/373 (90.6%)	291/338 (86.1%)	47/338 (13.9%)	

^*^Variables with p < 0.2 which were included in the multivariate analysis

^a^P value calculated by comparing those with invasive ductal carcinoma to other categories combined

^b^P value calculated by comparing in situ and stages 1/2 vs stages 3, 4 and recurrence

**Table 4:** Logistic regression model predicting *BRCA1/2* P/LP variant from demographic, clinical and test indication data in study participants

Outcome: *BRCA1/2* P/LP variant	Regression coefficient	Std Error	*Z* value	*P* value	OR (95% CI)
Intercept	− 2.17	0.32	− 6.772	0*	0.11 (0.06–0.21)
Family history reported	0.68	0.32	2.13	0.033^*^	1.97 (1.05–3.70)
> 1 primary BC	0.82	0.43	1.88	0.060	2.26 (0.92–5.15)
Late cancer stage	− 0.45	0.30	− 1.48	0.138	0.64 (0.35–1.17)
Black African	0.95	0.35	2.67	0.008^*^	2.58 (1.28–5.18)
White	0.38	0.42	0.92	0.357	1.47 (0.62–3.25)

^*^Indicates significant values

The model showed that family history and ancestry were independently associated with the presence of a P/LP variant in *BRCA1/2*. Participants with a family history were almost twice as likely to have a P/LP variant in *BRCA1/2* (OR 1.97; 95% CI 1.05–3.70, *p* = 0.03), while participants of Black African ancestry had 2.58 (95% CI 1.28–5.18, *p* < 0.01) times higher odds of having a P/LP variant than the reference group (those of Mixed Ancestry). From the regression analysis, we calculated that participant of Black African ancestry reporting a family history had a 5.01 times higher chance of having a P/LP variant in *BRCA1/2* compared to the reference (Mixed Ancestry participants without a family history).

**Fig. 1:**
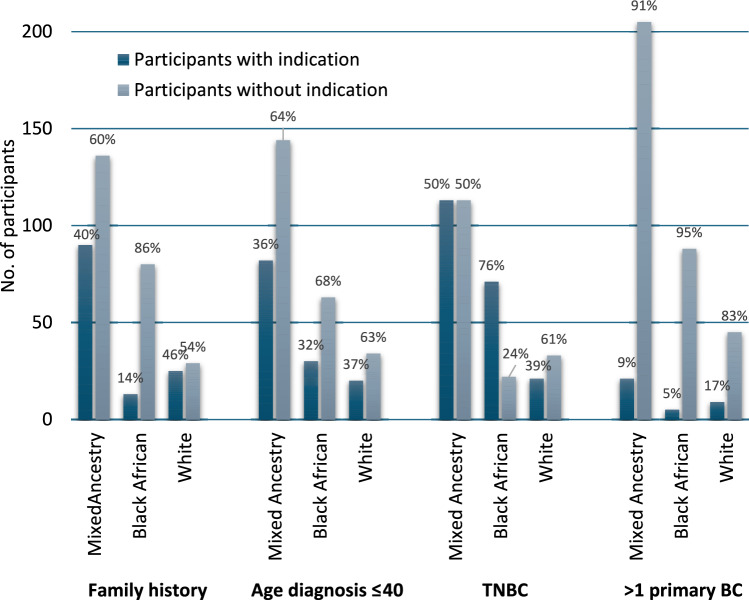
Frequency of NDOH genetic testing criteria met in South African Mixed Ancestry, Black African and White ancestry groups. Testing criteria included family history, age of diagnosis ≤40 years, triple negative breast cancer (TNBC) and >1 primary breast cancer (BC)

Given that ancestry was a significant variable in the logistic regression analysis, we compared the frequency of the four testing indications by ancestry (Fig. 1). The indications TNBC (*p* = 0.77) and > 1 primary BC (*p* = 0.08) were found to occur at similar frequencies across the three ancestry groups. However, participants of Black African ancestry were significantly less likely to meet the NDOH family history criterion (*p* < 0.001), and more likely to meet the age at diagnosis criterion (*p* < 0.001). Participants from all three ancestry groups were equally likely to meet one (*p* = 0.62), two (*p* = 0.73) or three test indications (*p* = 0.34).

## Discussion

The existence of a guideline for genetic testing demonstrates that the South African Department of Health acknowledges the benefit of identifying women with P/LP variants in BC susceptibility genes [36]. Our finding that almost 20% of participants meeting the recommended criteria had a P/LP variant detected indicates a high detection rate and suggests that these guidelines are useful in our setting. The higher prevalence of P/LP variants in *BRCA1/2* found in our study (15.4%) compared to other studies (9.2% and 6.0%) which investigated prevalence in women meeting different versions of the NCCN guidelines, may be due to the more stringent criteria specified in the NDOH guideline [37, 38]. Although, expanding genetic testing to the entire BC population would be ideal to optimise detection, limited resources in the country make this unachievable [39]. These resources include not only genetic testing, but also genetic counselling and changes to clinical management for those testing positive. Therefore, we recommend prioritizing the expansion of genetic counselling and testing services and the development of specific guidelines tailored to local needs as an equitable way to increase identification of women with P/LP variants.

Family history of cancer, age at diagnosis, TNBC and > 1 primary BC on their own were associated with a similar prevalence of P/LP variants in *BRCA1/2.* However, as has been demonstrated in other studies, participants with two or three indications were significantly more likely to have a P/LP variant in *BRCA1/2* detected [37, 38, 40]. This confirms that the criteria have a cumulative effect, each contributing to the likelihood of a P/LP variant, thereby making risk stratification possible. Although the NDOH guidelines were developed to detect *BRCA1/2* P/LP variants, clinically actionable variants in other BC susceptibility genes were found in an additional 5% of participants, supporting the utility of extending testing beyond *BRCA1/2* in our setting. However, the similar prevalence of P/LP variants among participants meeting one versus two or three of the criteria suggests that these criteria are ineffective at differentiating gradations of risk for variants in BC genes other than *BRCA1/2*. This finding is consistent with reports that the NCCN guidelines are less effective at identifying P/LP variants in moderate-risk BC susceptibility genes [11, 13, 41]. Further evaluation of the guidelines is needed to determine the proportion of all P/LP variants in women with BCs that are detected by applying the NDOH criteria.

We found that when controlling for other factors, reporting a family history and being of Black African ancestry were independently associated with having a P/LP variant in *BRCA1/2.* Family history is a well-established risk factor for a *BRCA1/2* P/LP variant. The reason proportionally more participants of Black African ancestry had a *BRCA1/2* P/LP variant is uncertain. One possibility is that *BRCA1/2*-related BC is more common in Black African women in South Africa, as found in case–control studies in West African and African American populations [4–6], although population studies have not been conducted locally. Alternatively, but not mutually exclusive, the proportion of *BRCA1/2-*related BC may be higher because environmental factors contribute less risk. This is compatible with South African registry data indicating that the prevalence of BC in the Black African population (lifetime risk 1 in 43) is lower than that of both Mixed Ancestry (1 in 18) and White (1 in 10) populations [42]. It is also consistent with evidence of increasing BC incidence in African countries, that has been attributed to “westernization” of lifestyles [43]. Population-based studies would be required to disaggregate these possibilities. A further possible cause of the disparity in *BRCA1/2* prevalence may be differences in the NDOH criteria met by Black African participants; they were less likely to meet the family history and far more likely to meet the age of diagnosis criterion. However, as age of diagnosis was not found to be associated with a P/LP variant any more strongly than the other criteria, but family history was, this seems an unlikely explanation.

The low proportion of participants of Black African ancestry meeting the family history criterion may be attributed to the above-mentioned lower incidence of BC or possibly to a lower penetrance of *BRCA1/2* P/LP variants, as was found in a case–control study in Ghana [4]. Additionally, health-related disparities, which disproportionately affect Black South Africans [44], may contribute to shorter life expectancy, reducing the likelihood of a cancer family history, given the positive association of cancer with age [45]. The disparity in reported family history may also be the result of a lower awareness of medical family history in Black African participants. This supposition is supported by findings from a study in the USA which showed that medically under-served groups (primarily African Americans and non-English speakers) had less knowledge of their family cancer history [46]. Likewise, a South African study found that 30% of their predominantly Black African participants with idiopathic dilated cardiomyopathy did not have knowledge of their family history. Reasons included having little recent contact with relatives living elsewhere, poor medical literacy and difficulty accessing relatives’ death certificates [47]. The low frequency of family history being reported by Black African women requires exploration. However, our finding suggests that ancestry should be considered when offering genetic testing for hereditary breast cancer. We therefore recommend that in our setting Black African women with BC who have any family history of hereditary BC-associated cancers should be offered genetic testing even if they do not meet the defined NDOH criteria. In addition, awareness of the medical family history should be promoted.

Our assessment of the NDOH guidelines was limited by the relatively small numbers of P/LP variants in demographic sub-groups and in genes other than *BRCA1/2*. Consequently, we could not determine the predictive value of the different indications for each ancestry group or gene. The NDOH criteria themselves are premised on prevalence and penetrance of P/LP variants being relatively similar to those in Western populations. Our study adds to evidence from studies elsewhere in Africa that this may not be the case. It is important to note that we assessed a clinical setting in which various genetic tests were performed depending on availability, cost and other factors. Targeted mutation analysis alone was performed in a minority of cases, and 13 participants were excluded because this was negative. Also, in those receiving sequencing, the number of genes sequenced varied. The quoted prevalence of hereditary breast cancer (19.9%) is therefore an estimate, but if all 13 excluded cases were included but negative, this would give a lower bound of prevalence of 19.3% (75/389).

The high prevalence of P/LP variants detected in participants as well as evidence that P/LP variant risk can be stratified by the number of criteria met, provides evidence of the value of the NDOH guidelines in a South African public health service setting. The increased detection rate of *BRCA1/2* P/LP variants in women with either a relevant family history or Black African ancestry, and especially both together is important to guide genetic testing and thereby is relevant to genetic counselling. Further research and possible refinement of the NDOH guidelines should be considered to address the fact that Black African women were less likely than other women to meet the family history criterion and more likely to meet the age of onset criterion.

## Supplementary Information

Below is the link to the electronic supplementary material.

Supplementary file1 (DOCX 19 KB)

## Data Availability

The datasets generated and analysed during the current study are not publicly available but are available from the corresponding author on reasonable request.
