# Supplementary material for: Application of genetic testing criteria for hereditary breast cancer in South Africa
Source: Breast Cancer Res Treat. 2025 Jan 7;210(2):477–86. doi: 10.1007/s10549-024-07585-3 (PMC11930871; doi:10.1007/s10549-024-07585-3)
Supplement: Supplementary file 1 — Supplementary file1 (DOCX 19 KB) [file 10549_2024_7585_MOESM1_ESM.docx]

**Supplementary Table 1: P/LP variants found in participants meeting the NDOH* testing criteria**

| Gene | P/LP Variant | Number in probands |
| --- | --- | --- |
| *ATM* | c.5228C>T (p.Thr1743Ile) | 1 |
|  | c.5279dup (p.Met1760Ilefs*9) | 1 |
|  | c.6139_6146del (p.Val2047*) | 1 |
|  | c.7271T>G (p.Val2424Gly) | 2 |
|  | c.8307G>A (p.Trp2769*) | 1 |
| *BRCA1* | c.1016dup (p.Val340Glyfs*6) | 1 |
|  | c.1360_1361del (p.Ser454*) | 1 |
|  | c.1398del (p.Lys467Argfs*8) | 1 |
|  | c.181T>G (p.Cys61Gly) | 1 |
|  | c.1953_1956del (p.Lys653Serfs*47) | 2 |
|  | c.2307_2313del (p.Ile769Metfs*21) | 1 |
|  | c.2641G>T (p.Glu881*) | 5 |
|  | c.4484G>T (p.Arg1495Met) | 1 |
|  | c.5096G>A (p.Arg1699Gln) | 1 |
|  | c.5153-1G>A (Splice acceptor) | 1 |
|  | c.66_67insA (p.Glu23Valfs) | 1 |
|  | c.66dup (p.Glu23Argfs*18) | 1 |
|  | Deletion (Exons 1-12) | 2 |
|  | Deletion (Exons 1-15) | 1 |
|  | Deletion (Exons 20-21) | 2 |
|  | Deletion (Exons 4-6) | 1 |
| *BRCA2* | c.6449_6450insTA ,p.Lys2150?fs | 1 |
|  | c.3865_3868del (p.Lys1289Alafs*3) | 1 |
|  | c.3881T>A (L1294*) | 1 |
|  | c.5771_5774del (p.Ile1924Argfs*38) | 12 |
|  | c.582G>A (p.Trp194*) | 3 |
|  | c.5946del (p.Ser1982Argfs*22) | 1 |
|  | c.6447_6448dup (p.Lys2150Ilefs*19) | 3 |
|  | c.7558C>T (p.Arg2520*) | 1 |
|  | c.7934del (p.Arg2645Asnfs*3) | 10 |
|  | c.8961_8964delGAGT (p.Ser2988Phefs*12) | 1 |
|  | c.9105T>A (p.Tyr3035*) | 1 |
| *CHEK2* | c.1100del (p.Thr367Metfs*15) | 1 |
|  | c.283C>T (p.Arg95*) | 1 |
|  | c.779del (p.Ser260*) | 1 |
| *PALB2* | c.2167_2168del (p.Met723Valfs*21) | 1 |
|  | c.2835-1G>C (Splice acceptor) | 3 |
| *RAD51C* | c.491_492del (p.Phe164Tyrfs*3) | 1 |
| *RAD51D* | c.619T>C (p.Ser207Pro) | 1 |
| *TP53* | c.742C>T (p.Arg248Trp) | 1 |
|  | c.831_835del (p.Cys277Trpfs*27) | 1 |

*NDOH – National Department of Health (South Africa)
